# Genomic and molecular analysis of conserved and unique features of soybean *PIF4*

**DOI:** 10.1038/s41598-018-30043-2

**Published:** 2018-08-22

**Authors:** Hina Arya, Mohan B. Singh, Prem L. Bhalla

**Affiliations:** Plant Molecular Biology and Biotechnology Laboratory, School of Agriculture and Food, Faculty of Veterinary and Agricultural Sciences, The University of Melbourne, Victoria, 3010 Australia

## Abstract

Phytochrome-interacting factor 4 (PIF4) participates in light signaling by interacting with photoreceptors, phytochromes, and cryptochromes. Although well characterized in *Arabidopsis*, PIF4′s role in crop plants is unknown. Here we performed the first integrated genomics, transcriptomics, and molecular characterization of PIF4 in soybean (*Glycine max*) plants. Fifteen identified *Glycine max* PIFs (GmPIFs) grouped into PIF3, PIF4, and PIF8 subfamilies based on their phylogenetic relationships. The GmPIF4 subfamily formed two distinct clades (GmPIF4 I and GmPIF4 II) with different amino acid sequences in the conserved bHLH region. Quantitative transcriptional analysis of soybean plants exposed to different photoperiods and temperatures indicated that all PIF4 I clade GmPIF4s conserved *PIF4*-like expression. Three out of four *GmPIF4* transcripts of the GmPIF4 I clade increased at 35 °C compared to 25 °C under short day conditions. RNA sequencing of soybeans undergoing floral transition showed differential regulation of *GmPIF4b*, and ectopic *GmPIF4b* expression in wild type *Arabidopsis* resulted in an early flowering phenotype. Complementation of *GmPIF4b* in *Arabidopsis pif4-101* mutants partially rescued the mutant phenotype. PIF4 protein levels peaked before dawn, and a GmPIF4b protein variant was observed in soybean plants treated at high temperatures.

## Introduction

Environmental factors such as light and temperature have a profound effect on plant physiology and development; not only their presence but also the duration of exposure^1^. The photoperiod (light and dark phase length) influences molecular signaling^2^, with the circadian clock synchronizing these environmental signals with endogenous rhythms to ensure optimal development and reproduction^2,3^.

High-throughput sequencing and genetic analyses have revealed that phytochrome interacting factors (PIFs), a class of basic helix-loop-helix (bHLH) transcription factors, play crucial roles in integrating photoperiodic signals through photoreceptor, phytochrome and cryptochrome, interactions. In the model plant *Arabidopsis thaliana*, PIFs belong to the bHLH superfamily of proteins, with the PIF subfamily consisting of PIF1, PIF3, PIF4, PIF5, PIF6, PIF7, and PIF8^4^. The bHLH domain contains a stretch of 50–60 amino acids that comprises two segments: a stretch of around 40 amino acids forming two amphipathic α-helices separated by a variable length loop and a 10–15 basic amino acid domain with DNA-binding capacity^5^.

PIF proteins have predominately been studied in *Arabidopsis* shade avoidance responses^6,7^. PIFs interact with the light-activated form of phytochromes (Pfr) through their highly-conserved active phytochrome-binding (APB) motifs^6^. PIFs typically accumulate in the dark, peak at dawn, and then degrade in the presence of light by interacting with Pfrs and ubiquitin-proteasome degradation^6^. PIF transcription is regulated by the evening circadian clock complex, with the ELF3-ELF4-LUX complex directly binding to *PIF4* and *PIF5* promoters to suppress their expression and regulate circadian responses^8^. It has recently been suggested that PIF4 acts as an integrating hub for light and temperature-related signals and the evening circadian clock-expressed factor TOC1 to regulate thermoresponsive plant growth^9^. PIF4 is also a central phytochrome regulator during *Arabidopsis* flowering under short day conditions^3^ through control of hormonal networks^10,11^. In *Arabidopsis*, PIF4 also controls auxin (indole acetic acid, IAA) signaling by modulating the expression of *SMALL AUXIN-UP RNA* (*SAUR*) genes at high temperatures^10^. PIF4 interacts with the blue light receptor *CYPTOCHROME 1* (*CRY1*) to regulate high temperature-mediated hypocotyl elongation by increasing IAA concentrations through stimulation of *YUC8* (*YUCCA8*) and *TRYPTOPHAN AMINOTRANSFERASE OF ARABIDOPSIS 1* (*TAA1*) gene expression^12^. PIF4 and PIF5 together play a crucial role in leaf senescence, activating *ETHYLENE INSENSITIVE 3* (*EIN3*), *ABSCISIC ACID INSENSITIVE 5* (*ABI5*), and *ENHANCED EM LEVEL* (*EEL*) gene expression to produce the senescence hormones ethylene and abscisic acid^13^. Clearly, PIFs have pleiotropic roles in model plants, but their roles in other plants of commercial value is less well characterized.

Soybean (*Glycine max* (L.) Merrill) is a leguminous crop that is mainly used as a source of protein and vegetable oil and that can fix atmospheric nitrogen via a symbiotic relationship with soil-borne microorganisms. The soybean genome is complex due to two genome duplication events estimated to have occurred 59 and 13 million years ago^14^. The paleopolyploid soybean genome presents the exciting opportunity to explore evolutionary diversification in gene function occurring due to chromosomal rearrangements during duplication. The presence of multiple forms/copies of a gene is often linked to the acquisition of new functions (neo-functionalization) or division of labor to divide the function (sub-functionalization) in a species. These gene diversification events lay the foundation for phenotypic variability and adaptability in plants^15^. Soybean flowering and pod set is dependent on the photoperiod^16^. Hence, soybean cultivars are divided into different maturity groups depending on day length requirements, and some of the quantitative trait loci that affect soybean flowering have recently been reported^17–19^.

The roles of PIFs and their interactions with phytochromes during soybean flowering have yet to be investigated. Moreover, the functions of genes related to temperature and light perception in soybean are unknown. The recent sequencing of the soybean genome has provided the means to examine the genes participating in soybean flowering pathways. To explore PIF4’s roles in soybean plants, especially short day-specific signaling in soybean flowering, we studied all the *GmPIF* sequences present in the soybean genome. Phylogeny, conserved protein motifs, and expression profiles of these genes were comprehensively analyzed using bioinformatics approaches. Further, gene expression patterns under flowering non-inductive (long day) and flowering inductive (short day) light conditions and at elevated temperatures were quantitatively analyzed. The function of differentially regulated *GmPIF4* (*GmPIF4b*) was studied by ectopic expression in *Arabidopsis* Col-0 and in *pif4-101* mutants. We reveal structural and functional divergence in soybean *PIF4* genes and proteins.

## Results

### Identification, phylogeny, and subcellular localization of *GmPIF* genes

Systematic and comprehensive database searches of the available genome sequences of leguminous plants revealed multiple *PIF* family members. To investigate the phylogenetic relationship between different PIFs and their evolutionary conservation, four leguminous plants with sequenced genomes were considered. The Phytozome search and phylogenetic analysis grouped fifteen PIF-like sequences into PIF4, PIF3, and PIF8 clades. There is strong evidence that soybean has undergone two whole genome duplication events during evolution. Based on the chromosomal evidence, soybean’s recent lineage-specific palaeotetraploidization was probably an allotetraploidy event^14^ preceded by an early legume duplication event occurring near the origins of the papilionoid lineage^20^. Recently, the Legume Family Working Group (LPWG) refined the classification of the Leguminosae family into six subfamilies: *Caesalpinioideae*, *Cercidoideae*, *Detarioideae*, *Dialiodeae*, *Duparquetioideae*, and *Faboideae*^21^, with soybean assigned to the family *Faboideae*.

**Figure 1:**
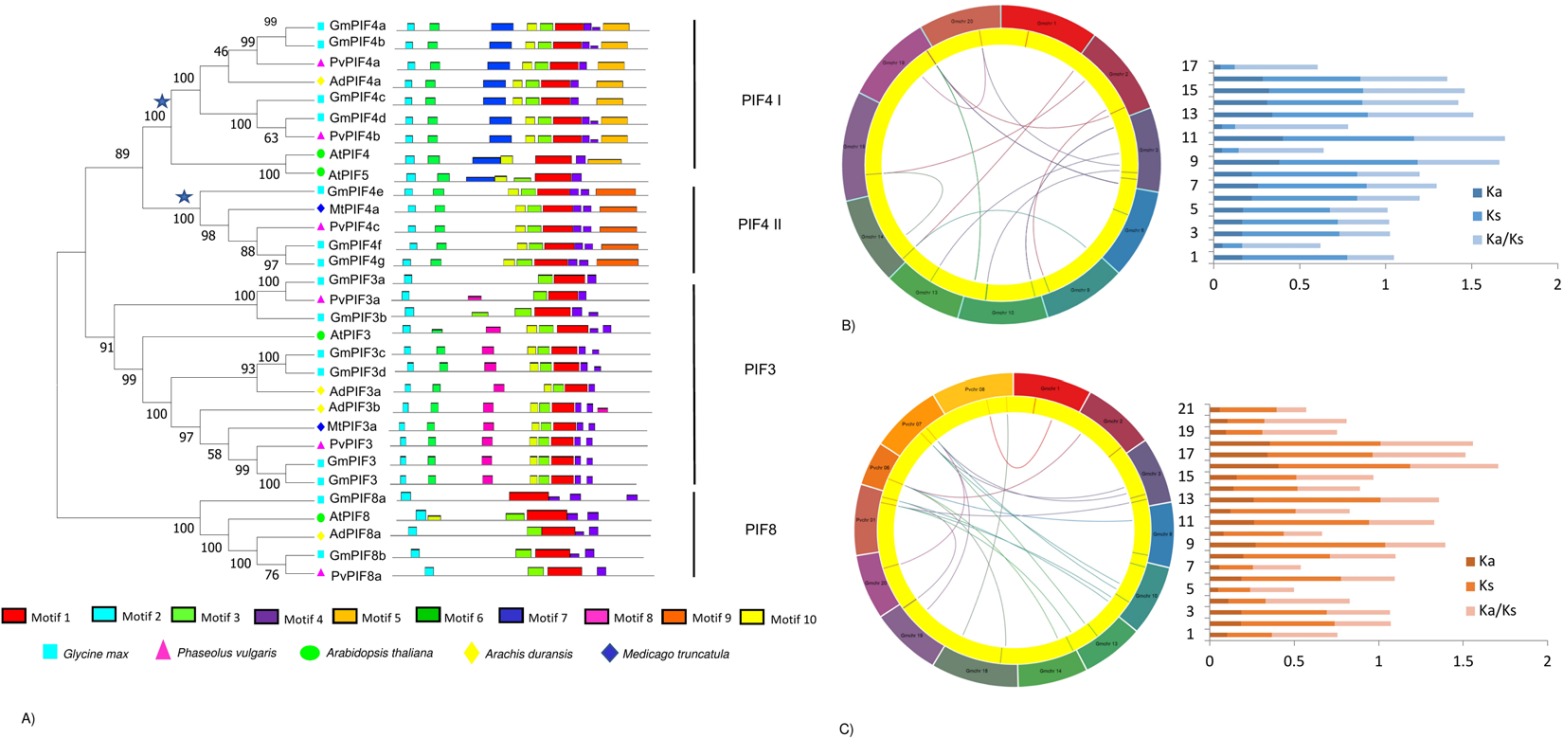
(**A**) Phylogenetic analysis and motif comparisons of three main PIF subfamilies in legumes. Protein sequences were aligned by Clustal W and trees constructed using the neighbor-joining method with 1000 bootstrap replicates in MEGA 7. Legume PIF protein sequences were used to predict the conserved motifs using the MEME Suite web server. Asterisks represent the GmPIF4 I and Gm PIF4 II clades. (**B**) Relationships of *Glycine max* PIFs. (Left) Genomic locations of PIFs and duplicated gene pairs in the *Glycine max* genome. (Right) Ka-Ks statistics of the duplicated gene pairs. (**C**) Relationships of *Glycine max* PIFs to *Phaseolus vulgaris* PIFs. (Left) Genomic locations of PIFs and duplicated gene pairs in the *Glycine max* and *Phaseolus vulgais* genome. (Right) Ka-Ks statistics of the related gene pairs. (B) and (C) generated using the Circa tool of omgenomics, http://omgenomics.com/.

To establish the phylogenetic relatedness of legume PIF proteins, soybean (*Glycine max*), common bean (*Phaseolus vulgaris*), barrel clover (*Medicago truncatula*), and peanut (*Arachis duranesis*) sequences were extracted. All these plants belong to the *Faboideae* family, with the soybean, common bean, and peanut short day plants and *Medicago* a long day plant^22^. Phylogenetic analysis using the neighbor-joining algorithm revealed that different soybean PIFs group into different clades (PIF4, PIF3, and PIF8; Fig. 1A) and include the signature PIF4 sequence of *Arabidopsis*. Soybean PIF4s grouped into two clades, GmPIF4 I and GmPIF4 II (marked with asterisks in Fig. 1A), with GmPIF4a, GmPIF4b, GmPIF4c, GmPIF4d grouping into GmPIF4 I and GmPIF4e, GmPIF4f, and GmPIF4g grouping into GmPIF4 II. Similarly, PIF3 and PIF8 were classified based on their relatedness to the signature PIF3 and PIF8 *Arabidopsis* sequences. Their position in the tree indicated that these multiple PIF copies in soybean may have evolved at different evolutionary points. Some of the PIF4s in soybean retain family-specific relatedness because of the early legume genome duplication event, while the other PIFs arose more recently due to a soybean-specific duplication event. PIFs grouped more closely to the common bean PIFs compared to *Medicago* and peanut, consistent with the common bean being a closer relative^21^.

### Analysis of GmPIF protein sequence motifs

Ten motifs were identified and designated motifs 1–10 (Supplementary Figure 1). Motif 5, 7, and 9 mainly distinguished GmPIF4 I from GmPIF4 II proteins (Fig. 1A). A lack of motif 9 and 5 and the presence of motif 8 was a characteristic feature of PIF3s. Furthermore, motifs 3, 5, 8, and 9 were absent in PIF8s. Motif patterns help to distinguish sequences, as motif location and frequency are important for protein folding during translation. Motifs also act as recognition sequences for molecules involved in important processes such as post-translation modifications, subcellular transport and localization, and translation start and termination^23^.

### Two whole genome duplications contributed to *GmPIF* gene family expansion

The genomic survey showed an uneven distribution of fifteen *GmPIF* genes on 11 soybean chromosomes (Fig. 1B). Chromosome 3 and 19 had two genes each, chromosome 10 had three genes, and the other nine genes were located on chromosomes 1, 2, 8, 13, 14, 18, and 20. Two main gene duplication types occur during evolution: tandem duplication, resulting in gene clusters; and segmental duplication, which gives rise to members scattered across the genome. 5,671 putative soybean transcription factor genes have been identified, of which 9.5% show tandem duplication^14^. Detailed analysis of *GmPIF* genes revealed that two gene pairs of the *PIF3* subfamily were tandemly duplicated (*GmPIF3a-GmPIF3c* and *GmPIFb-GmPIF3f*; Fig. 1B).

We next estimated the possible duplication time according to their pairwise distances (*Ks* values) based on previous soybean studies^14^. *Ks* values of 0.06–0.39 correspond to the 13 million years ago (Mya) *Glycine* lineage-specific genome duplication, Ks values of 0.40–0.80 correspond to the 59 Mya early legume whole genome duplication, and *Ks* values greater than 1.5 mostly correspond to the most ancient gamma event^14^. Based on this, four *GmPIF* pairs were associated with 13 Mya *Glycine*-lineage specific duplications and 13 pairs were associated with 59 Mya early legume duplication (Supplementary Table 2). *Ka/Ks* calculations were also performed to estimate the selection pressure on *GmPIF* sequences, which indicated that all *GmPIFs* were subjected to purifying selection pressure (Fig. 1B; Ka/Ks = 1, neutral selection, Ka/Ks < 1, purifying selection, and Ka/Ks > 1 positive selection. Purifying selection results in the selective removal of deleterious alleles^24^.

Finally, we investigated duplication blocks between the soybean *PIF* genes and its close relative the common bean *Phaseolus vulgaris*. Fourteen *GmPIF* genes formed putative orthologous relationships with four *PvPIF* genes. All showed Ka/Ks values < 1, indicating purifying selection (Fig. 1C). Duplication events played a significant role in the expansion of the legume *PIF* gene family.

### bHLH domain alignment shows conserved and unique amino acid residues in GmPIFs

**Figure 2:**
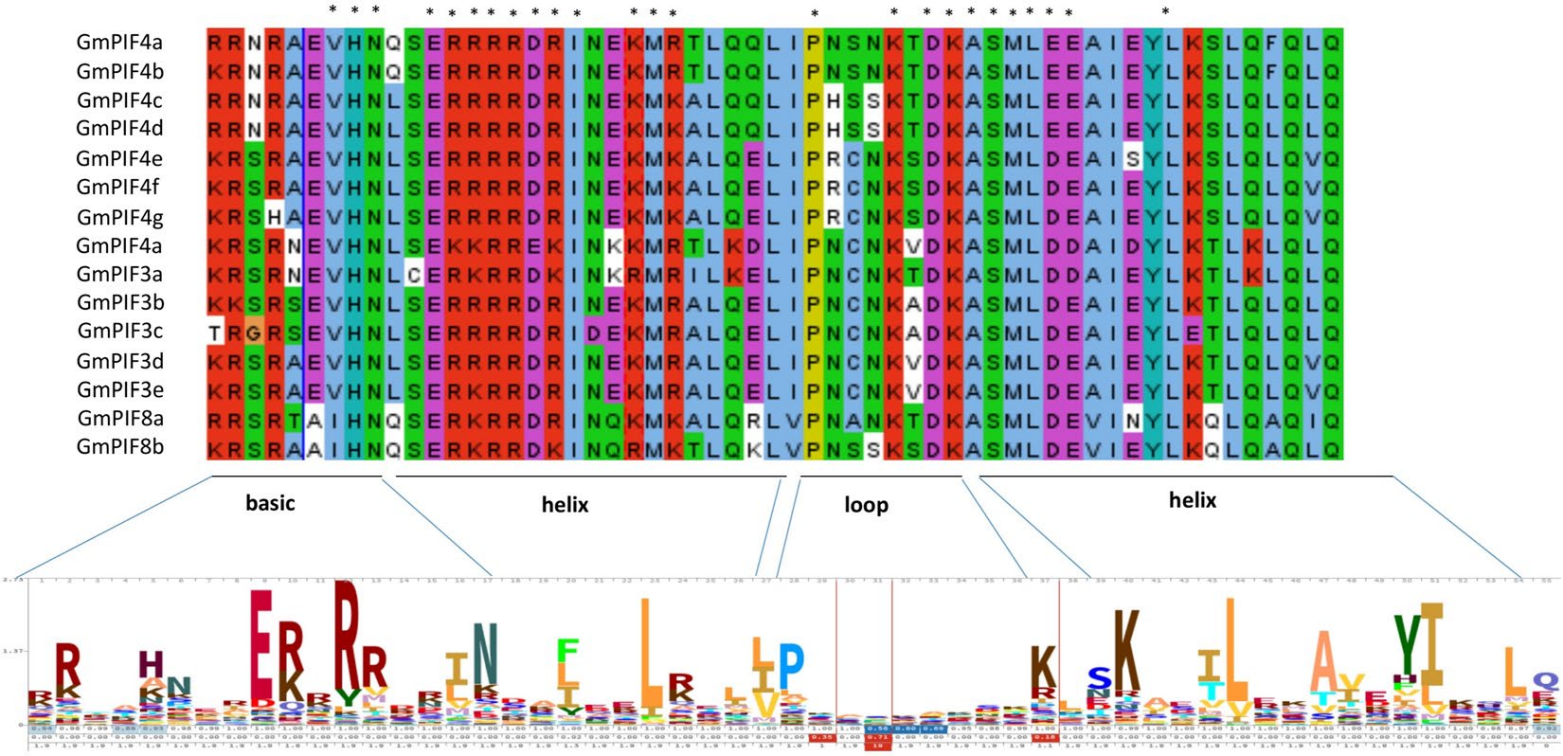
Clustal alignment of the bHLH region of all soybean PIFs. The alignment was generated using Jalview 2.10.3. Amino acid residues were color coded according to Clustal’s default color scheme. Unconserved residues are white. Consensus logo for bHLH was taken from Pfam (ID PF00010), http://pfam.xfam.org/family/PF00010#tabview=tab4.

Protein sequence alignment revealed the presence of the highly conserved bHLH domain in all soybean GmPIF proteins. Plant bHLH proteins bind to their target sequences at G-box (5′-CACGTG-3′) motifs, a subset of the E-box motif (5′-CANNTG-3′). This binding event is characterized by contact of glutamic acid residue (E) at position 9 of the basic stretch with the CA nucleotides of the E/G box^25,26^. While the E residue at position 5 of the basic stretch was conserved in all GmPIF4s and GmPIF3s, it was replaced by alanine (A) in GmPIF8s. Alignment also indicated the presence of different amino acid residues in the basic region of the bHLH domain, with the basic amino acid arginine (R) conserved in all GmPIFs except GmPIF4g (replaced with histidine (H). Another difference was the presence of asparagine (N) in GmPIF4a, GmPIF4b, GmPIF4c, and GmPIF4d but replaced by serine (S) and glycine (G) in the remaining soybean PIFs. These differences in basic region amino acids allow the bHLH proteins to discriminate their target DNA (Fig. 2)^27^.

### Differential response of *GmPIFs* during floral transition and transcript abundance in different soybean tissues

**Figure 3:**
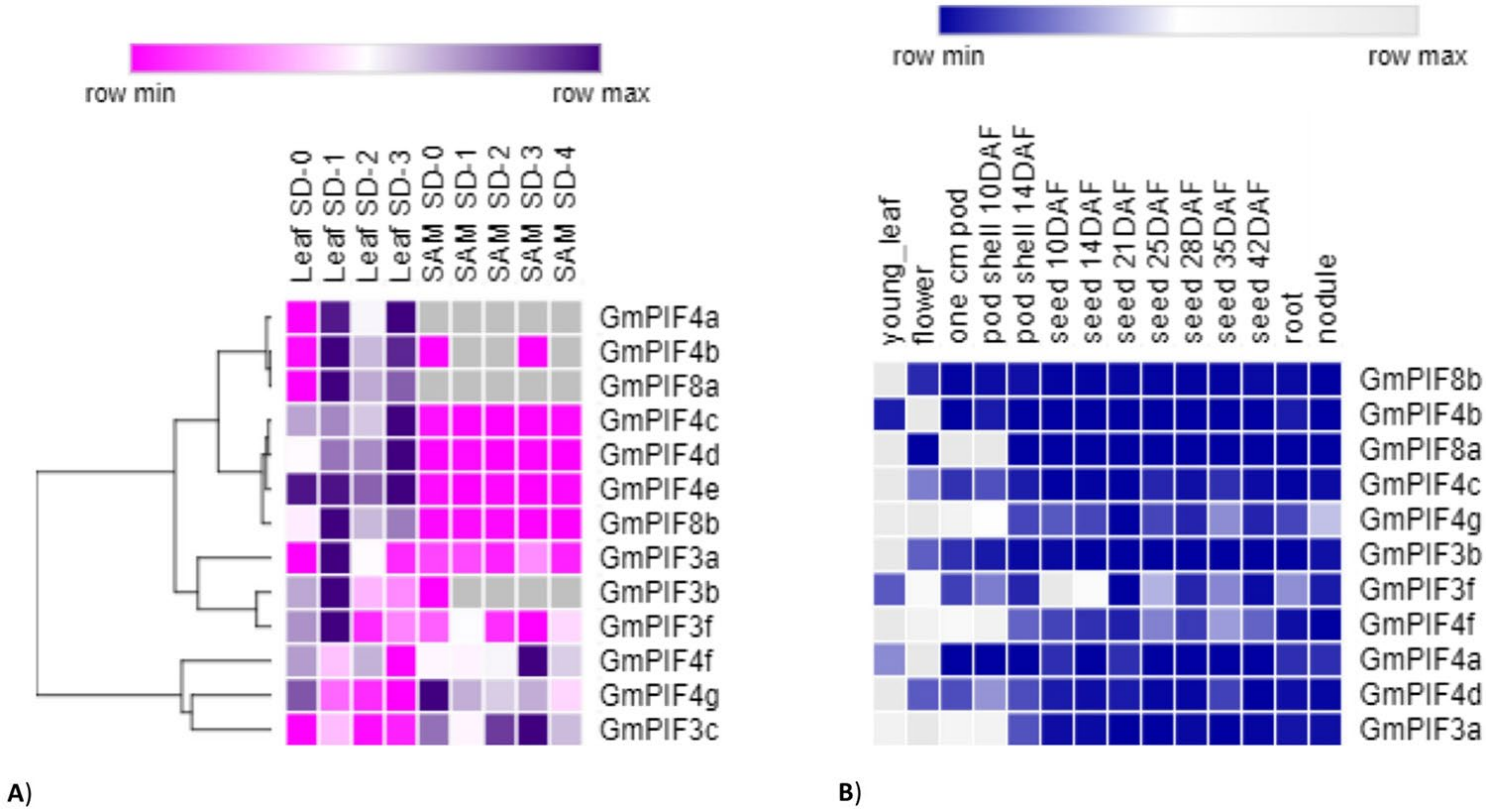
The expression profiles of *PIF* genes in: (**A**) soybean leaf and shoot apical meristem (SAM) undergoing floral transition. Samples were derived from short day-0 (SD-0) to short day-4 (SD-4). Values are in RPKM. A gene was considered differentially expressed if it showed significant changes at one time point as compared to the previous time point (Leaf SD1-Leaf SD0, Leaf SD2-Leaf SD1 Leaf SD3-Leaf SD2, SAM SD1-SAM SD-0, SAM SD2-SAM SD-1, SAM SD3-SAM SD-2, SAM SD4-SAM SD-3; (**B**) Expression of soybean *PIFs* in different tissues at every growth stage. Data were obtained from the SoyBase RNA expression atlas in the form of gene expression counts of the uniquely mappable reads (https://www.soybase.org/soyseq/tables_lists/tablesearch.php). Heat maps were constructed using Morpheus (https://software.broadinstitute.org/morpheus/).

The RNA sequencing analysis for floral transition was performed using plants grown for ten days in long photoperiod and then exposed for one day to short photoperiod (SD) for floral induction. Leaf and shoot apical meristem (SAM) samples were collected at SD-0, SD-1, SD-2, and SD-4. 13/15 *GmPIFs* were differentially regulated during floral transition. *GmPIF4f*, *GmPIF4g*, and *GmPIF3c* were abundantly expressed in the SAM, while *GmPIF4a-e* were expressed in the leaves. *GmPIF3a*, *b*, and *f* were previously shown to be highly regulated in leaves^28^. Tissue-specific expression analysis showed that *GmPIF8b*, *GmPIF3b*, *GmPIF4c*, *GmPIF4d* were expressed in leaves, while *GmPIF8a*, *GmPIF4g*, *GmPIF4f*, *GmPIF3a* were present in leaves, flowers, and young pods. *GmPIF3f* was the only transcript observed in seeds, and no *GmPIF* transcript was observed at late developmental stages (Fig. 3)^29^.

### Long day specific diurnal rhythm of *GmPIF4* transcripts

**Figure 4:**
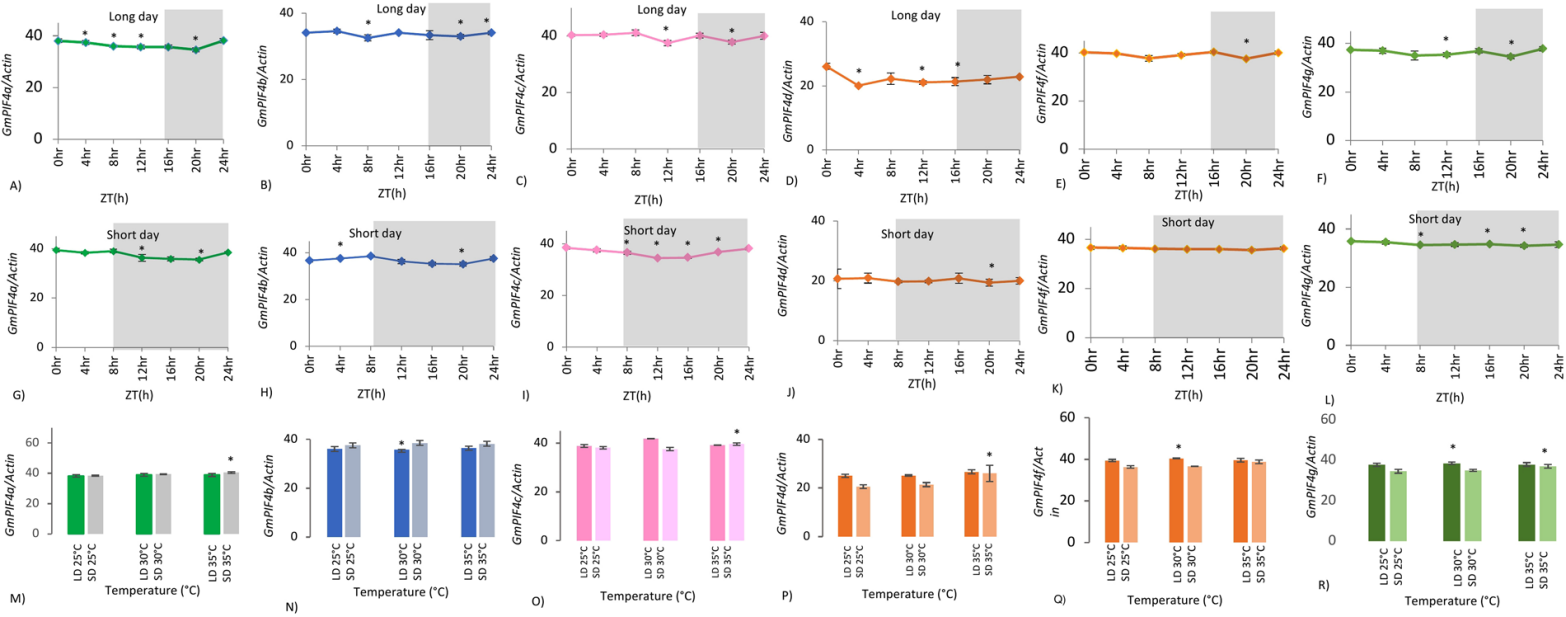
Expression of *GmPIF4a-d,f,g* transcription factors under different photoperiods and temperatures. (**A–F**) The expression levels of mRNA transcripts tested at different time points (0, 4, 8, 12, 16, 20 and 24 h) under long day photoperiod conditions. (**G–L**) The expression levels of mRNA transcripts tested at different time points (0, 4, 8, 12, 16, 20 and 24 h) under SD photoperiod conditions. (**M–R**) The expression levels of mRNA transcripts tested at three different temperatures (25 °C, 30 °C, and 35 °C) under both long day and SD photoperiods. Data are from three independent biological experiments, and error bars indicate standard deviations. Significant differences between data were calculated using Student’s *t*-test (all time points have been compared to time point of 0 h). Significant differences are indicated with asterisks (*), P < 0.05. Intervals on X-axis represent time points/temperature points and intervals on Y-axis represent expression levels calculated by the 40-ΔCt method. The shaded area of the graph represents night. At 0 h, samples were collected in the dark, just before the day began (dawn), and for temperature experiments all samples were collected just before the day began (dawn).

Soybean leaves were sampled every four hours to examine whether soybean *PIF4s* were diurnally regulated. Over long days, all transcripts showed differential responses during the day and night. During light periods, *GmPIF4b* transcript abundance significantly declined at 8 h compared to 0 h. *GmPIF4c* and *GmPIF4g* showed significant decreases at 12 h. However, these transcripts were re-expressed in the last four hours of the day, i.e., between 12 and 16 h (Fig. 4A–F), consistent with the long day behavior of *Arabidopsis thaliana PIF4* transcripts, which re-accumulate on prolonged exposure to light and indicating that decreases in *PIF4* levels upon light exposure are transient^6^. *GmPIF4a* and *GmPIF4d* did not show typical *PIF4* like expression in long day photoperiod.

### Short day specific diurnal rhythm of *GmPIF4* transcripts

Samples were collected every 4 hours to study *PIF4* transcription patterns after one short day treatment. One short day treatment was sufficient to alter the expression of *GmPIF4a*, *GmPIF4b*, and *GmPIF4c* (Fig. 4G–I), which accumulated during the dark (just before the day breaks), consistent with previous reports on the expression of *PIF4* in *Arabidopsis* during short days^30^. However, *GmPIF4f* and *GmPIF4g* showed no diurnal fluctuations under short day conditions (Fig. 4K,L). Often, duplication events silence the function of an ancient gene, with selective pressure giving rise to homologs with new functions^14^.

### Expression of *GmPIF4* transcripts at different temperatures under long and short day conditions

A temperature-dependent role for PIF4 in flowering and blue light responses in *Arabidopsis* has been reported^12^. To investigate how soybean *PIF4* genes respond to temperature under flower-inducing short photoperiod conditions, the expression levels of soybean *PIF4* transcripts were analyzed at 25 °C, 30 °C, and 35 °C under long and short-day conditions. There were no significant changes in transcript levels under long day conditions except for *GmPIF4f and GmPIF4g*, which showed an increase at 30 °C compared to at 25 °C (Fig. 4M–R). However, under short day conditions, *GmPIF4a*, *GmPIF4c*, *GmPIF4d*, and *GmPIF4g* transcripts significantly increased at 35 °C compared to 25 °C (Fig. 4M–R). According to the thermosensory activation model of flowering in *Arabidopsis*, PIF4 integrates short day photoperiod signals and combines them with the ambient temperature signal^30^ under the control of the endogenous clock. Kumar *et al*.^30^ proposed that, at higher temperatures, PIF4 directly interacts with *flowering locus T* (*FT*, florigen) to activate the flowering pathway in *Arabidopsis*. Further, temperature-based changes in *PIF4* transcripts are rate limiting for the biological response, because H2A.Z nucleosomes decrease the accessibility of PIF4 to the *FT* promoter at cool temperatures^30^. Since soybean is a warm climate plant requiring short day conditions for floral induction, the increase in *GmPIF4* transcript abundance at 35 °C (short day) indicates a possible role for soybean *PIF4s* in high temperature-mediated initiation of flowering.

### Ectopic expression of *GmPIF4b* in *Arabidopsis* Col-0 plants

**Figure 5:**
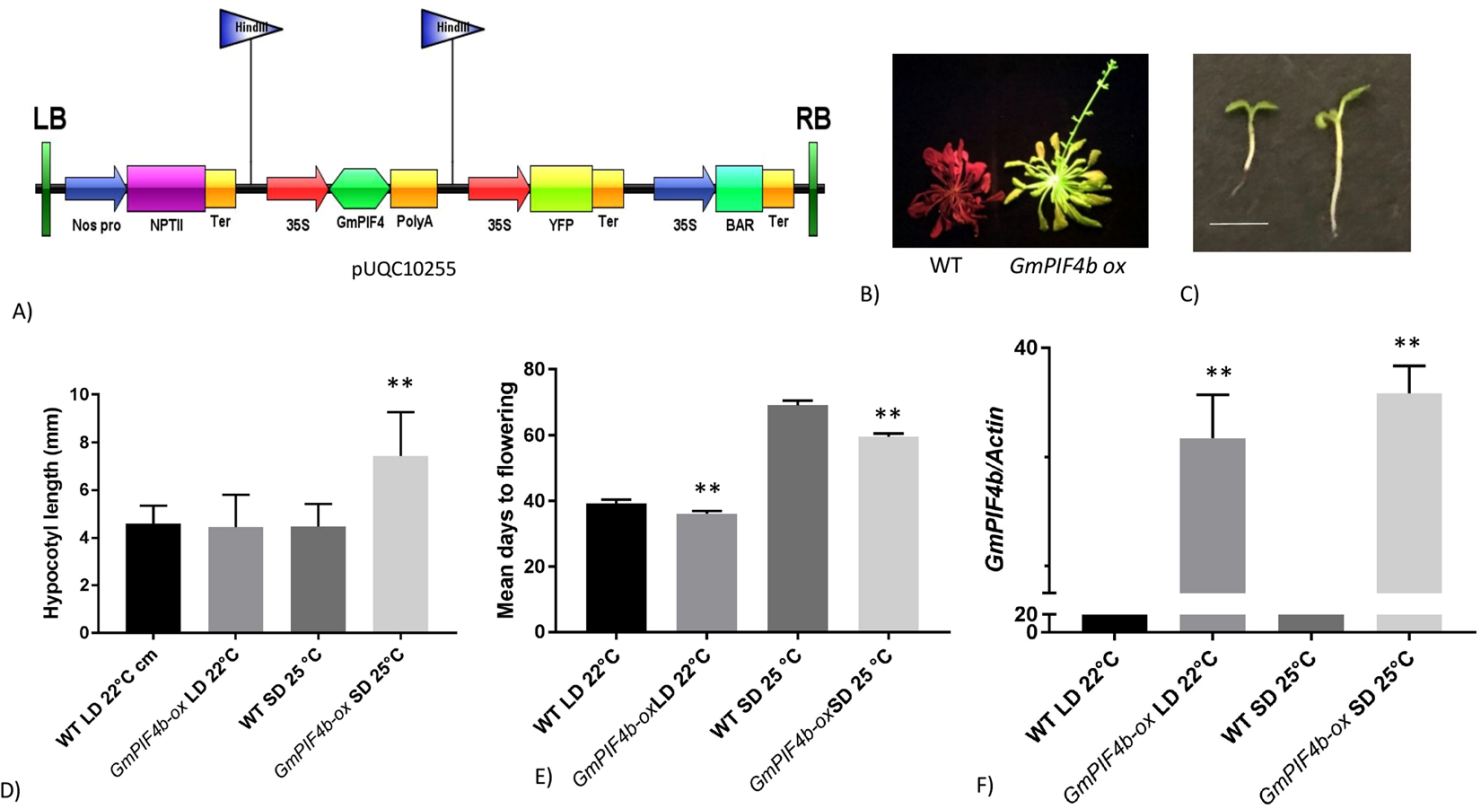
Overexpression analysis of *GmPIF4b* in Col-0 *Arabidopsis* background. (**A**) Binary vector construct containing *GmPIFb* CDS driven by the *35s* promoter. (**B**) Early flowering phenotype in *GmPIF4b-ox* lines at SD 25 °C, (C) Hypocotyl length phenotype in *GmPIF4b-ox* lines at SD 25 °C. (**D**) Measurement of hypocotyl length of different lines. (**E**) Mean days to flowering of different lines. (**F**) Expression levels of *GmPIF4b* in WT and *GmPIF4b-ox* lines normalized against *Arabidopsis actin* gene. The significant differences between data were calculated using Student’s *t*-test. Significant differences are indicated with asterisks: (*)P < 0.05 and (**)P < 0.01. Error bars represent SD, n = 10. WT plants appear orange because they were observed in blue light.

Analysis of RNA-seq data of soybean plants undergoing floral transition showed that *GmPIF4b* was differentially regulated in leaves. Hence, to further characterize gene function, *GmPIF4b* was expressed ectopically in *Arabidopsis* Col-0 plants and trangenic lines studied under long day 22 °C and short day 25 °C conditions. Transgenic lines had longer hypocotyls at SD-25 °C and flowered 8–10 days earlier than wild type lines under short day conditions. However, expression of *GmPIF4b* had no effect under long day conditions, indicating a conserved function for *GmPIF4* (Fig. 5)^31^.

### Complementation of *GmPIF4b* in the *Arabidopsis pif-101* mutant background

**Figure 6:**
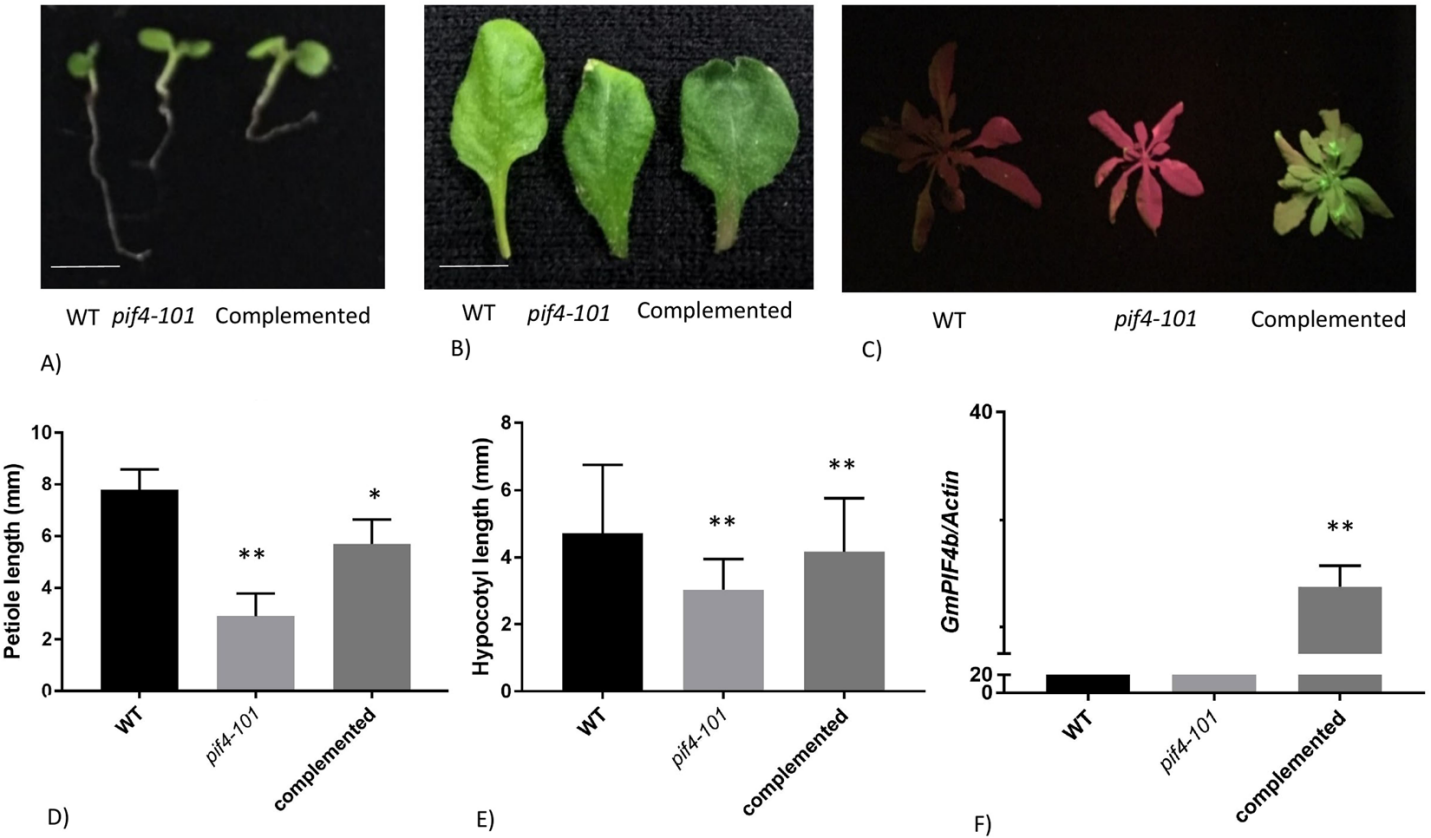
Mutant complementation analysis *of GmPIF4b* in *pif4-101 Arabidopsis* background. (**A**) Comparison of hypocotyl lengths in WT, *pif-101*, and complemented lines. (**B**) Petiole length phenotype in WT, *pif-101*, and complemented lines at SD 25 °C. (**C**) Rosette size phenotype in WT, *pif-101*, and complemented lines. (**D**) Measurement of petiole length in different lines. (**E**) Measurement of hypocotyl length in different lines. (**F**) Expression levels of *GmPIF4b* in WT, *pif4-101*, and complemented lines normalized against *Arabidopsis actin* gene. The significant differences between data were calculated using Student’s *t*-test. Significant differences are indicated with asterisks: (*)P < 0.05 and (**)P < 0.01. Error bars represent SD, n = 10. WT plants appear orange when observed in blue light.

*pif4-101* mutants have a T-DNA insertion in exon 5 of the *Arabidopsis PIF4* gene. These mutant plants have shorter hypocotyls in the dark and a compact rosette (reduced petiole length) phenotype^6^. We transformed the *pif4-101 Arabidopsis* mutant with the 35S::*Gmpif4*::*polyA* construct for a gain-of-function analysis. Hypocotyl length was recorded in seedlings grown. Furthermore, petioles were also measured to assess rosette size. *GmPIF4b* partially rescued the mutant phenotype for both hypocotyl and petiole lengths under short day 25 °C conditions. Petiole length was almost 8 mm in wild-type, 2.6 mm in *pif4-101*, and 6 mm in complemented lines (Fig. 6) and hypocotyl length in complemented lines was 0.86 times of the WT (Fig. 6).

### GmPIF4b protein levels peak four hours before dawn under both long and short-day conditions

**Figure 7:**
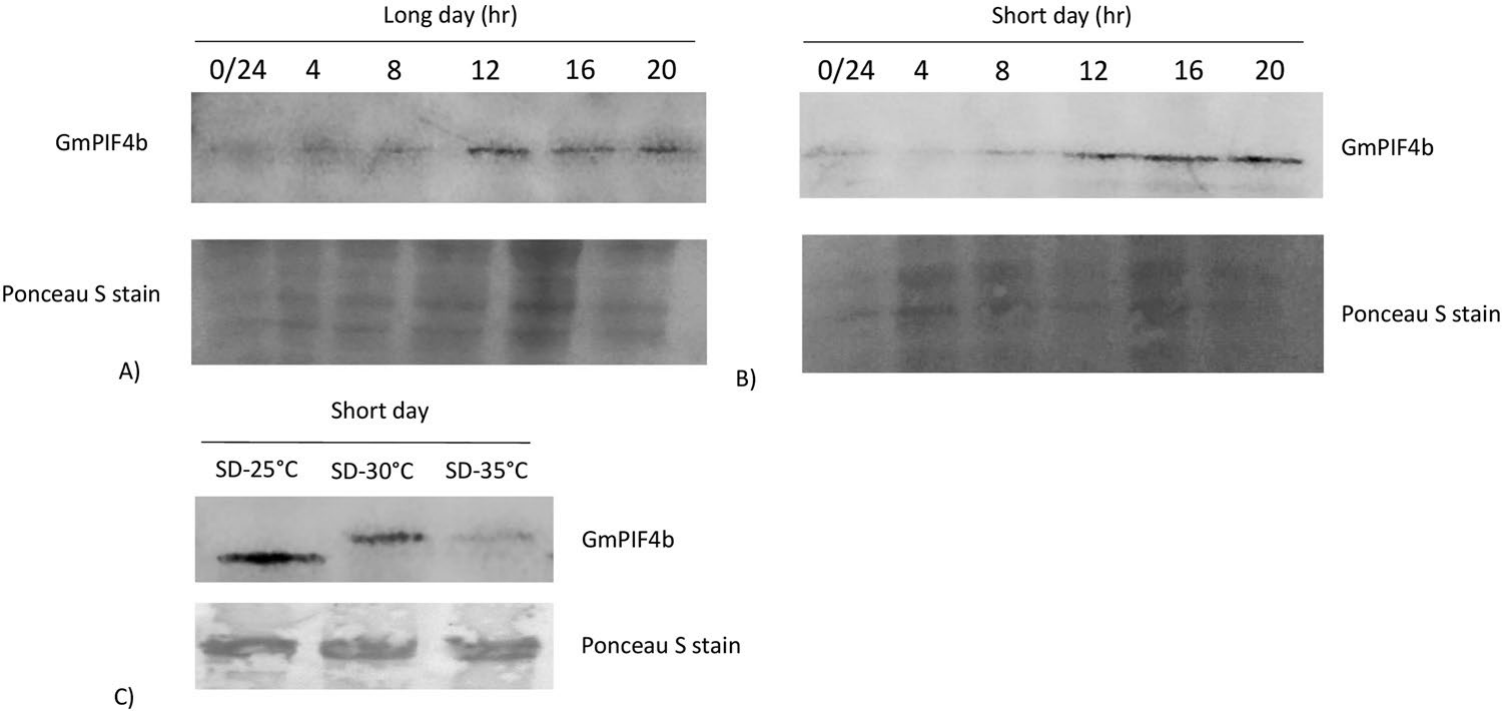
Immunoblots showing GmPIF4b protein dynamics under different photoperiods and temperatures. (**A**) Diurnal rhythm of GmPIF4b under long day conditions. Samples were taken (every four hour) after eleven long days. (**B**) Diurnal rhythm of GmPIF4b under short day conditions (every four hours). Samples were taken from ten-day-old plants with fully expanded leaves after one short day treatment. (**C**) GmPIF4b expression at different temperatures. Equal amount of total nuclear protein (1.5 ug) was loaded in all experiments to compare the GmPIF4b levels. Full length blots are shown in Supplementary Figure 3. Two independent biological replicates were performed.

To study the diurnal rhythm of PIF4 protein, protein expression was assessed every 4 h under long and short day conditions. *GmPIF4b* transcript was more abundant in the leaves of the plants grown under short day conditions compared to long day conditions. However, *GmPIF4b* transcripts followed a strict diurnal rhythm under both photoperiod conditions. For both conditions, protein levels peaked four hours before dawn (Fig. 7). Arabidopsis PIF4 levels are known to peak during the night due to superimposition of the clock and photoperiodic pathways^3^, and PIF4 is thought to be under the control of the evening complex. Further, the TOC1 component of the clock binds to PIF4 in the evening and inactivates it  in *Arabidopsis*^9^. Here, the GmPIF4b protein expression rhythm in soybean was similar to *Arabidopsis*. GmPIF4b protein also showed the highest expression in soybean leaves at SD-1, suggesting involvement in floral transition. RNA-seq studies have previously indicated major reprogramming during floral transition, especially when SAM converts from the vegetative to reproductive stage after 4–6 short day treatment^32^.

### GmPIF4b variant observed at elevated temperatures show unique temperature adaptations in soybean

*Arabidopsis* lines containing the 35S::PIF4:HA construct have been reported to contain slightly higher PIF4 protein levels at 27 °C than at 12 °C and 22 °C^30^. To study the effect of temperature on PIF4 protein expression, soybean plants were treated with a range of temperatures (25 °C to 35 °C), reflecting soybean as a warm temperature crop with ambient temperatures for soybean growing at different latitudes often exceeding 30 °C. A different molecular weight variant form of GmPIF4b was observed following exposure to plants at higher temperature. (Figure 7C). Higher molecular weight variant observed in response to higher temperature might reflect a protein modification that merits further experimental evaluation.

## Discussion

Soybean is a major leguminous crop used to produce a significant amount of vegetable oil and protein for human consumption and fodder for animals. Soy products are increasingly used as meat and milk substitutes globally. Hence, the demand for breeding high-yield varieties of this commercially important crop in our changing environment is increasing. To refine yields, a full understanding of the key regulators of flowering and development is essential. PIF4 is a bHLH transcription factor that is thought to act as an integrating hub for light and temperature signals in *Arabidopsis*. However, its role in important crops such as soybean, a paleopolyploid, has yet to be investigated.

Two gene duplication events occurred in the soybean genome nearly 59 and 13 million years ago, which were followed by gene diversification, loss, and numerous chromosomal rearrangements leading to 75% of soybean genes being present as multiple copies^14^. Here we extracted fifteen *GmPIF* transcription factor genes from the Phytozome database and compared their sequences at both the nucleotide and amino acid levels. *GmPIFs* could be grouped into three significant subfamily clades (GmPIF4, GmPIF3, and GmPIF8) based on their conserved protein sequences. *GmPIF4* could be further divided into two groups, GmPIF4 I and GmPIF4 II, based on sequence motif organization. This sequence-level observation supports the hypothesis that these transcription factors have undergone significant changes during evolution. Overall, there are estimated to be 31,264 gene paralogs in soybean, which may have developed from substitution and transversion events^14^.

PIF transcription factors use their bHLH domain to bind DNA and regulate their downstream targets. Our detailed comparison of this conserved domain for all the *GmPIF* protein sequences highlighted amino acid variations within the bHLH domains of these proteins. These variations in conserved domains suggest that it is likely that these transcription factors have different protein binding specificities.

Gene duplication analysis of the *GmPIF* family revealed that *GmPIF* genes expanded during both *glycine* lineage-specific and early legume duplication events nearly 13 Mya and 59 Mya, respectively. Synteny of *GmPIFs* with common bean (*Phaseolus vulagris*) *PvPIFs* was also evaluated to study the selective pressure on these genes, which showed that the Ka/Ks ratios for all *Gm-Pv* gene pairs were below 1, confirming purifying selection pressure.

Gene duplication serves as a mechanism to increase functional diversity^33^. In a paleopolyploid plant such as the soybean, these duplication events often lead to divergent expression patterns of closely related genes^34^. We found that the expression of these transcription factors varied in response to photoperiod and temperature stimuli. In *Arabidopsis thaliana*, *PIF4* transcription has been studied under both short day and warm conditions^30^. Soybean is a facultative short-day plant requiring the warm temperatures for floral initiation. Hence, we focused on studying *GmPIF4* transcription under short day conditions, under which four *GmPIF4s* showed similar expression to *Arabidopsis PIF4*, i.e., peaking at the end of the night phase (at dawn). All four GmPIF4s belong to the GmPIF4 I group; however, two GmPIF4s belonging to the GmPIF4 II clade did not follow a typical diurnal rhythm. A coincidence model has been proposed to understand short day-specific flowering in *Arabidopsis*, where PIF4 accumulates at the end of the night on short days due to coincidence between the internal (circadian rhythm) and external (photoperiod) cues^3^. During the light phase, PIF4 interacts with phytochromes and is degraded to switch on phytochrome signaling-mediated downstream processes^7^. In soybean, short days promote a shift from the vegetative to reproductive phase and hence control flowering^32^. Our data on GmPIF4 I group transcription is consistent with the co-incidence model, thus pointing towards conservation of gene function. To confirm this, ectopic expression of *GmPIF4b*, differentially regulated during soybean floral transition (*GmPIF4b*) in *Arabidopsis* Col-0 plants resulted in longer hypocotyls and an early flowering phenotype under short day 25 °C conditions, and partially recovered the phenotype of hypocotyl length and compact rosette in *Arabidopsis pif4-101* mutants.

Protein expression of GmPIF4b peaked four hours before dawn under both long photoperiod and short period conditions, indicating superimposition of the biological clock in controlling GmPIF4 expression in soybean plants. A unique GmPIF4 higher molecular weight variant was observed following treatment of soybean plants at higher temperatures, indicating involvement of post-translational modifications in regulating GmPIF4b protein levels at the high temperatures.

Hence, apart from the general functions of PIF4 in plants, this protein may participate in novel legume-specific development and function in soybean plants. Further detailed interaction analyses and metabolomic and proteomic-based studies are needed. Functional analysis of individual *PIF4* genes would uncover their specific roles in soybean development. This study paves the way for future research into specific biological functions of GmPIF4s in soybean development and floral transition.

## Methods

### Identification, phylogenetic analysis and sub-cellular localization prediction of soybean PIF family

*PIF* genes were searched by using the keywords of “PIF”, “Phytochrome Interacting factors”, and blast searches against *Arabidopsis* PIFs in the proteome database of the latest version of soybean genome (Wm82.a2.v1) in Phytozome. Subsequently, all the sequences with E-value below 0.01 were kept and checked for the presence of conserved basic helix loop helix (bHLH) by using Hidden Markov Model (HMM) profile (PF00010) in Pfam database, http://pfam.xfam.org/. Self-blast was performed on the resulting sequences list, and all the redundant sequences were removed. Similarly, PIF sequences for other legumes such as common bean (*Phaseolus Vulgaris*), barrel clover (*Medicago truncatula*) and peanut (*Arachis duransis*) were also searched. The resulting sequences were listed in a table (Supplementary Table 1) and aligned using ClustalW program with default parameters in the alignment window of MEGA7 software, http://www.megasoftware.net/ (Kumar, Stecher, and Tamura 2015). A phylogenetic tree was constructed using the PIF sequences of all the legumes and *Arabidopsis* using a neighbor-joining algorithm, JTT model, and partial deletion parameters. Based on the phylogenetic analysis, the putative soybean *PIFs* were named according to their respective clades. The subcellular localizations of *GmPIF* genes were predicted using LOCALIZER tool of the Commonwealth Scientific and Industrial Organization of Australia (CSIRO)^35^.

### Conserved protein motif search

MEME search (http://meme-suite.org/tools/meme) was used for protein motif search comparison^36^. The length of the motif was fixed to 6–100 amino acids. To detect motifs ZOOPS model was used, which considers that the motif occurrence can be zero or 1 in a sequence. Maximum 10 motifs were searched.

### Analysis of chromosome distribution, gene duplication and synteny with common bean

The chromosome distribution of soybean *PIF* genes was obtained from Phytozome, and duplicated genes were obtained from Plant genome duplication database (PGDD) (http://chibba.agtec.uga.edu/duplication/) by downloading the dataset of duplicated blocks in soybean genome^37^. Duplicated *PIF* gene pairs were searched in the dataset, and their nucleotide non-synonymous (*Ka*) to synonymous (*Ks*) ratios (Ka/Ks) was also calculated (Supplementary Table 2). *Ks* values were used to estimate the duplication time for soybean *PIFs*. Similarly, syntenic blocks between soybean and common bean were also searched. PGDD uses BLASTP to search for potential anchors (*E* < 1e-5, top 5 matches) between every possible pair of chromosomes in the genomes considered. Input for MCscan synteny search tool is the homologous pairs^38^. The built-in scoring scheme for MCscan is *min* (−log_10_*E*, 40) for every matching gene pairs and -1 for each 10 kb distance between anchors, similar to DAGchainer synteny tool^39^ and blocks that have scores >200 are kept. The resulting syntenic chains are evaluated using a procedure in ColinearScan and *E*-value < 1e-10 as a significance cutoff. The data for duplicated *PIF* gene pairs within soybean and their putative orthologs in common bean is listed (Supplementary Table 2).

### Multiple sequence alignment of the bHLH domain *GmPIFs*

The bHLH domain was identified after aligning the sequences of all 15 PIFs by using clustal alignment option in Jalview software^40^. The logo of bHLH domain was obtained from Pfam database of protein HMMs^41^.

### RNA seq data analysis for soybean undergoing floral transition and expression in different tissues

The RNA seq data for the soybean undergoing floral transition was obtained from previously published research (Wong *et al*. 2013). RNA sequencing data for the expression of *GmPIFs* in different tissues was obtained from soybase https://www.soybase.org/^29^. The RNA sequencing reads have been listed in Supplementary Table 3. The heat maps were constructed using MORPHEUS tool of the Broad Institute (https://software.broadinstitute.org/morpheus/).

### Plant material, Treatments and Expression analysis using qRT-PCR

For photoperiod-dependent expression analysis, two sets of (*Glycine max* [L.] Merill) cv. Bragg plants were grown for 10 long days (16hrs light, 8hrs dark) at 25 °C, 400 µMm^−2^s^−1^ light intensity. On 11^th^ day, one set of plants was subjected to one short-day (8hrs light, 16hrs dark) treatment. Leave samples (from three different plants within a set) from both sets were harvested every 4 hours for 24 hours.

For temperature dependent expression analysis, six sets of the plants were grown for 10 days under long day conditions. On the 11^th^ day, three sets were subjected to long-day at 25 °C, 30 °C and 35 °C and the other three sets were subjected to short-day at 25 °C, 30 °C and 35 °C respectively. Samples were collected at the end of the night. All the expression analyses were performed using three biologically replicated experiments.

Total RNA was extracted from the leaves samples by using Trizol method and cDNA was synthesized by using Superscript III Reverse transcriptase of Invitrogen. SYBR-Green master mix from Agilent Technologies was used. The expression data of *GmPIF4a*, *GmPIFb*, *GmPIF4c*, *GmPIF4d*, *GmPIF4f* and *GmPIF4g* transcripts was normalized against the expression of *Glycine max Actin* gene (*Glyma*.*08G146500*.*1*)^42^. Supplementary Figure 4 shows that this *actin* gene is not regulated diurnally or in response to heat treatment.

### *GmPIF4b* over-expression construct, *Arabidopsis* transformation, and transgenic line analysis

Total RNA was extracted from Soybean’s leaf tissue. The amplified DNA was cloned downstream of constitutive 35S promoter and resulting *35S::GmPIF4b::ployA* was used for plant transformation. *Arabidopsis* plants (wild type and mutants) were grown in soil, long day photoperiod and at 22 °C for 4 weeks (till flowering started). The first inflorescence was cut-off to promote flowering on lateral branches because we followed floral dip method for *Arabidopsis* transformation^43^. *Arabidopsis* seeds obtained from T0 generation were grown for 7 days and on 8^th^ day, these were sprayed with the herbicide Glufosinate (Basta) to select transgenic lines. Strong YFP signal was observed in the surviving plants (observed in blue light). This generation (T1) was examined for the expression of *GmPIF4b* by qRT-PCR. Similarly, complemented lines were obtained by infecting *Arabidopsis pif4-101* mutants with *GmPIF4b* over-expression construct. Hypocotyl lengths were analysis using Image J software (114 pixels were scaled to 1 cm).

### Production of rabbit polyclonal antibody against the GmPIF4b protein, plant nuclear protein extraction, and immunoblotting

The codon optimized GmPIFb gene construct by the GenScript Services (Hong-Kong) was used to express this protein in *E*.*coli*. The recombinant protein was quantified on a BSA standard curve and used for immunization of rabbits. The antibody was purified from total sera (Supplementary Figure 2). Nuclear protein was extracted according to Haerizadeh *et al*.^44^. For immunoblotting, 1.5 µg of total nuclear protein from soybean, primary antibody (anti-GmPIF4b developed in our lab) and secondary antibody (anti-rabbit IgG) were used. The blots were imaged using *Licor* western blot imager (800 nm channel). Two independent exeriments were performed to check the validity of the western blots. Dot blot analysis of *Arabidopsis* transgenic lines containing over-expressed GmPIF4b showed reactivity with GmPIF4b antibody, whereas wild type *Arabidopsis* (Col-0) showed no reactivity (Supplementary Figure 6).

### Data availability

Data described in this study can be obtained from the corresponding author by request.

## Electronic supplementary material

Supplementary materials

Supplementary Dataset 1

Supplementary Dataset 2

Supplementary Dataset 3

Supplementary Dataset 4
