# Supplementary material for: Genomic and molecular analysis of conserved and unique features of soybean PIF4
Source: Sci Rep. 2018 Aug 22;8:12569. doi: 10.1038/s41598-018-30043-2 (PMC6105606; doi:10.1038/s41598-018-30043-2)
Supplement: Supplementary file 1 — Supplementary materials [file 41598_2018_30043_MOESM1_ESM.pdf]

Supplementary Materials

Genomic and molecular analysis of conserved and unique features of soybean *PIF4*

Hina Arya, Mohan B Singh and Prem L Bhalla*

Plant Molecular Biology and Biotechnology Laboratory, School of Agriculture and Food, Faculty of Veterinary and Agricultural Sciences, The University of Melbourne, Victoria 3010, Australia

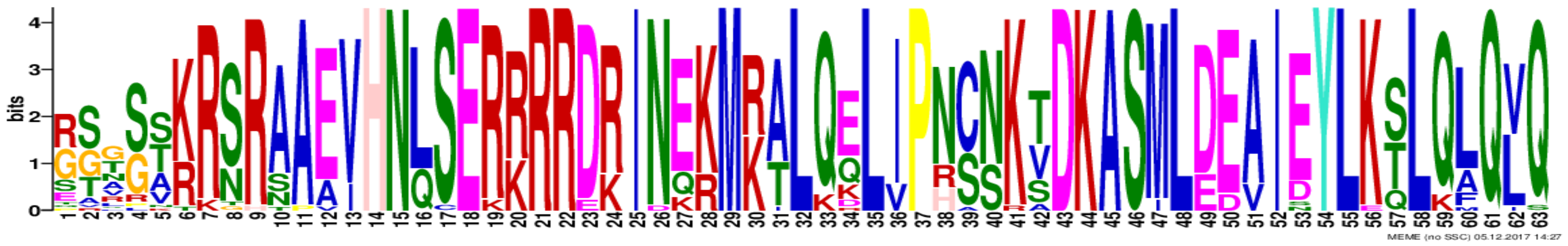

A) Motif 1

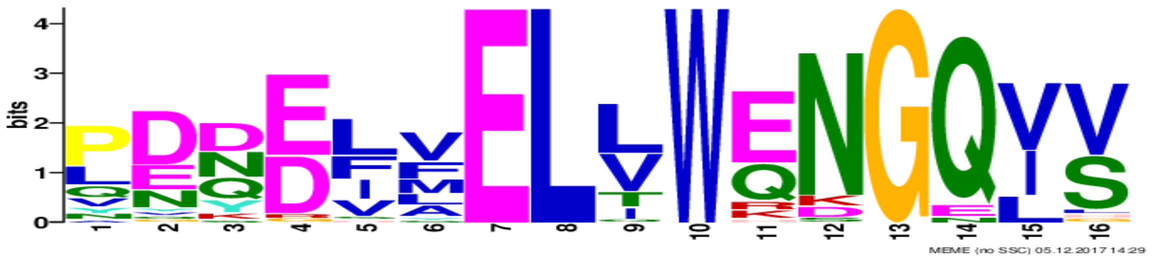

B) Motif 2

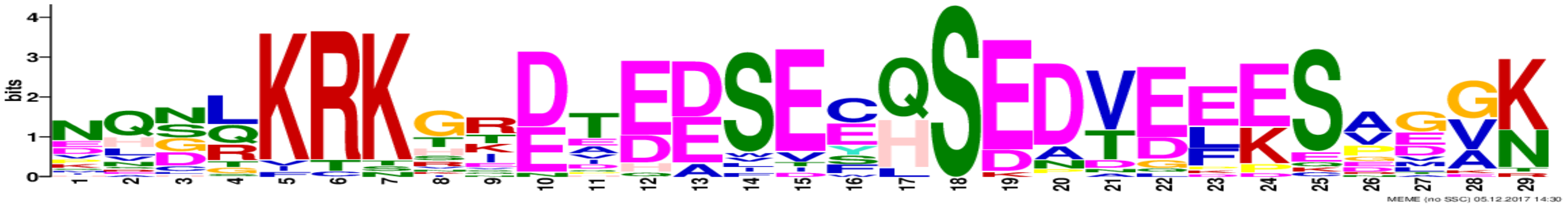

C) Motif 3

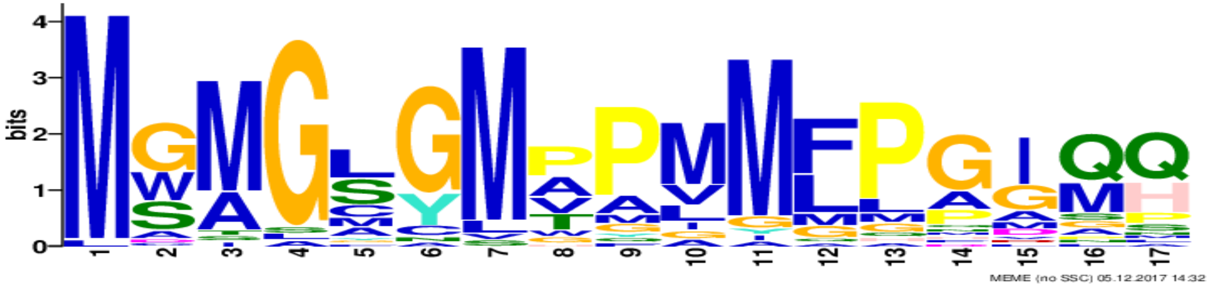

D) Motif 4

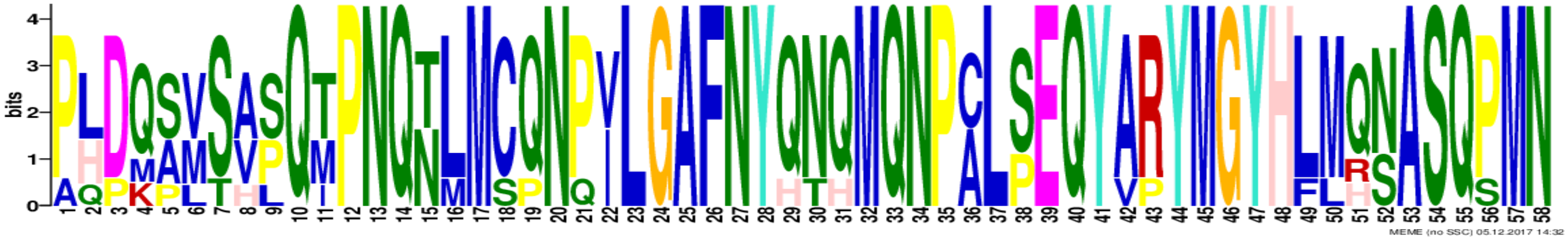

E) Motif 5

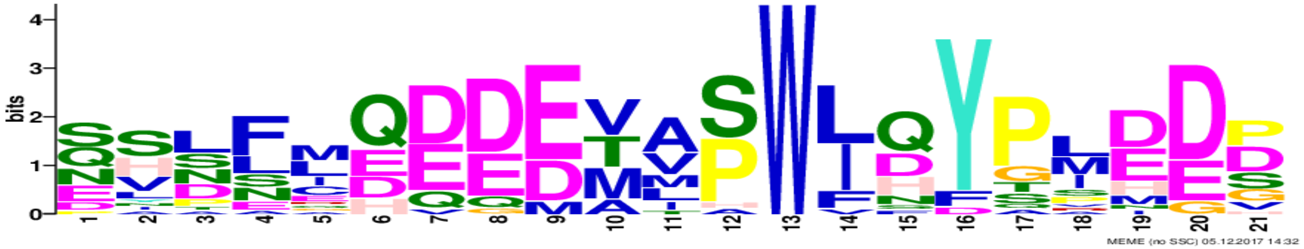

F) Motif 6

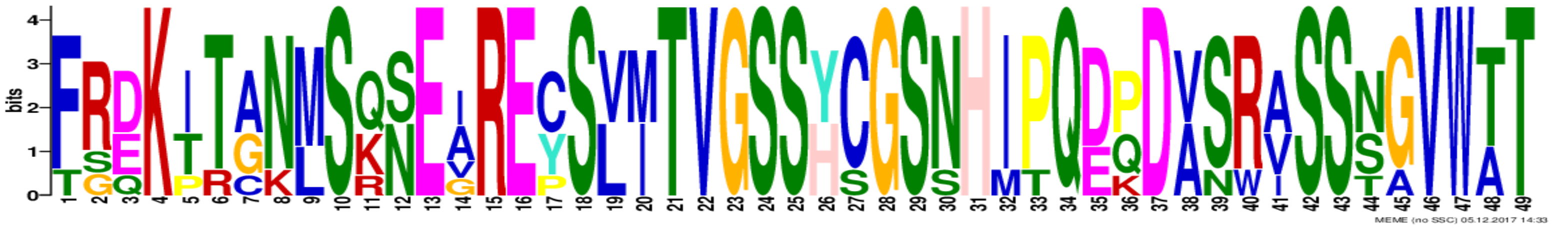

G) Motif 7

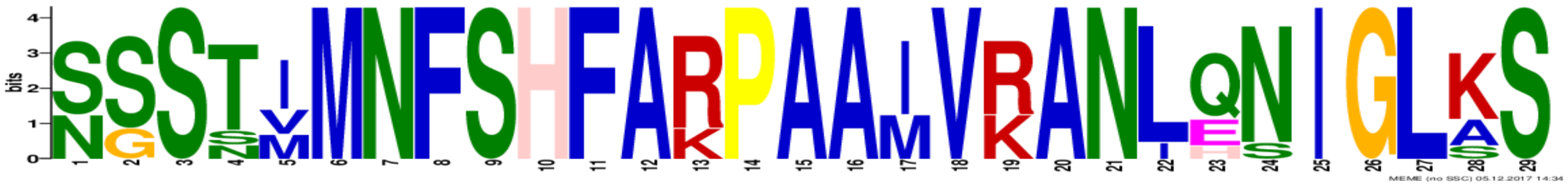

H) Motif 8

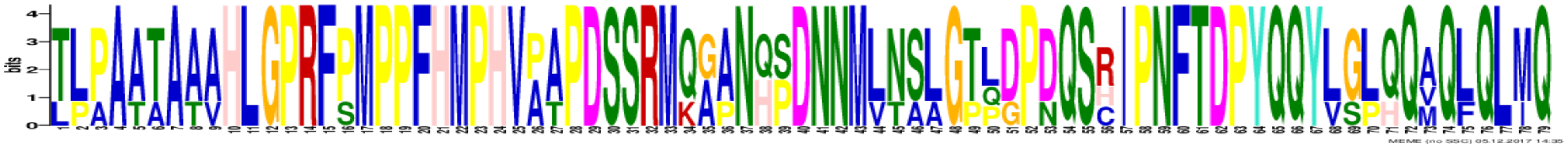

I) Motif 9

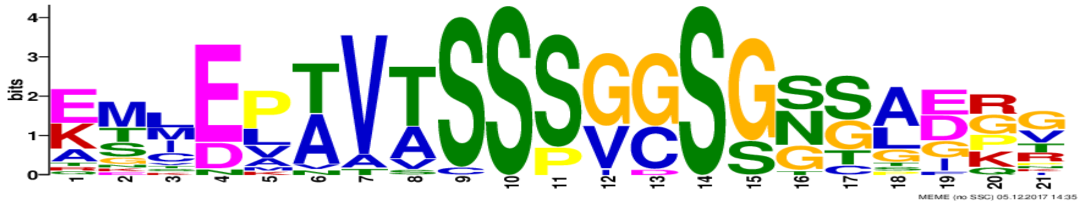

J) Motif 10

Supplementary Figure 1. A-J) Motif logos

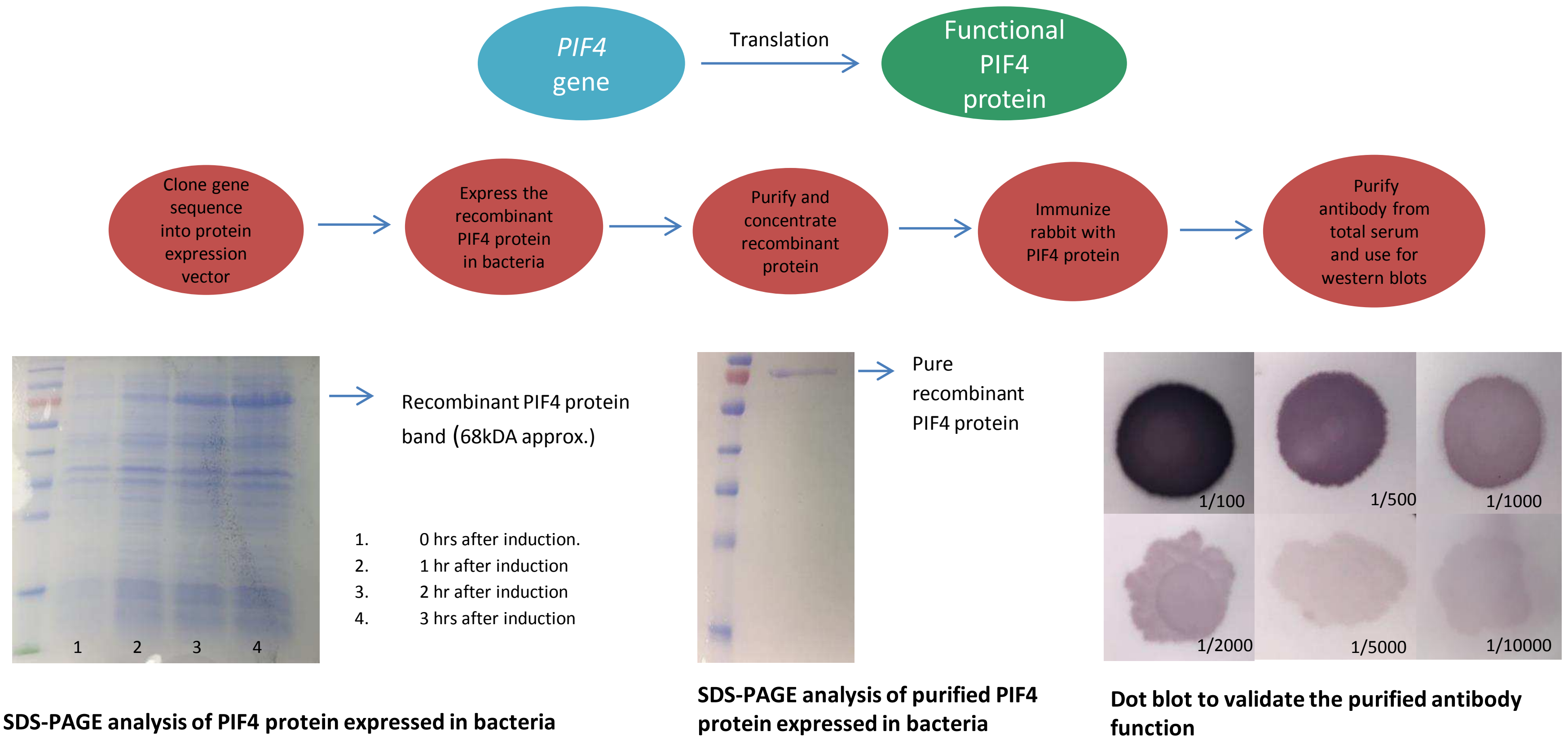

Supplementary Figure 2. Production of polyclonal antibody against GmPIF4b of soybean

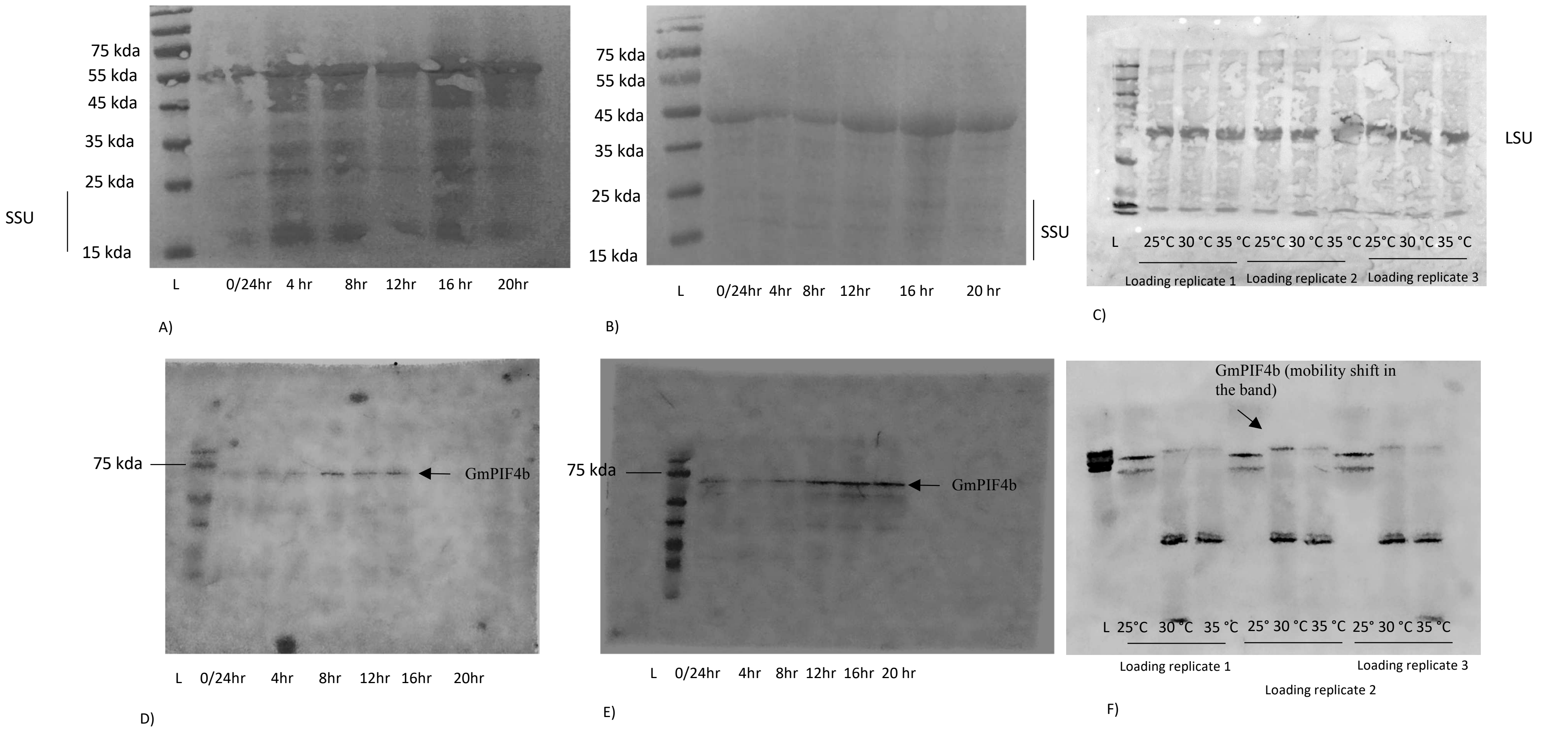

Supplementary Figure 3. Total protein ponceau S stains and western blots- (A) Total nuclear protein extracted every four hour from 11 day old soybean plants grown under long day conditions, SSU; small subunit of RuBisCO **(B)** Total nuclear protein extracted every four hour after one short-day treatment of plants that were grown in long-day conditions for 10 days **(C)** Total nuclear protein extracted from 10 day old plants that were exposed to one short-day at 25 °C, 30 °C and 35°C, LSU; large subunit of RuBisCO **(D)** Western blot to detect diurnal rhythm of GmPIF4b in long days. **(E)** Western blots to detect diurnal rhythm of GmPIF4b after exposure one short day. **(F)** Western blot to detect GmPIF4b at 25 °C, 30 °C and 35°C. Equal amount of total protein was loaded to compare the GmPIF4b levels in every experiment.

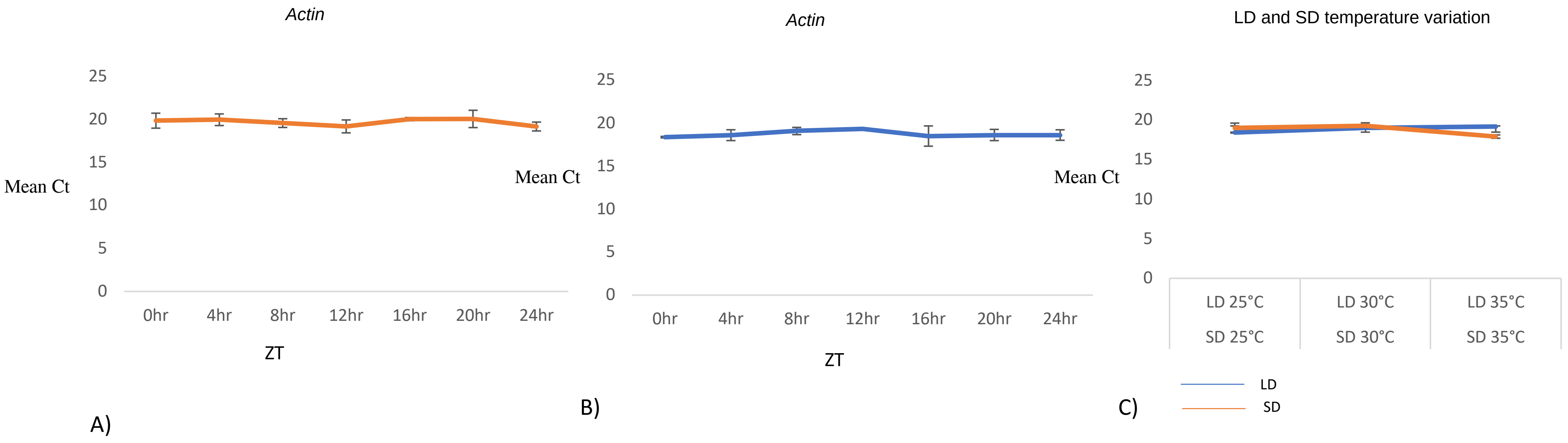

Supplementary Figure 4. A) Mean Ct (threshold cycle) values of *actin* gene from qRT-PCR study of soybeans treated in long photoperiod. B) Mean Ct (threshold cycle) values of *actin* gene from qRT-PCR study of soybeans treated in short photoperiod. C) Mean Ct (threshold cycle) values of *actin* gene from qRT-PCR study of soybeans treated at 25 °C, 30 °C and 35 °C.

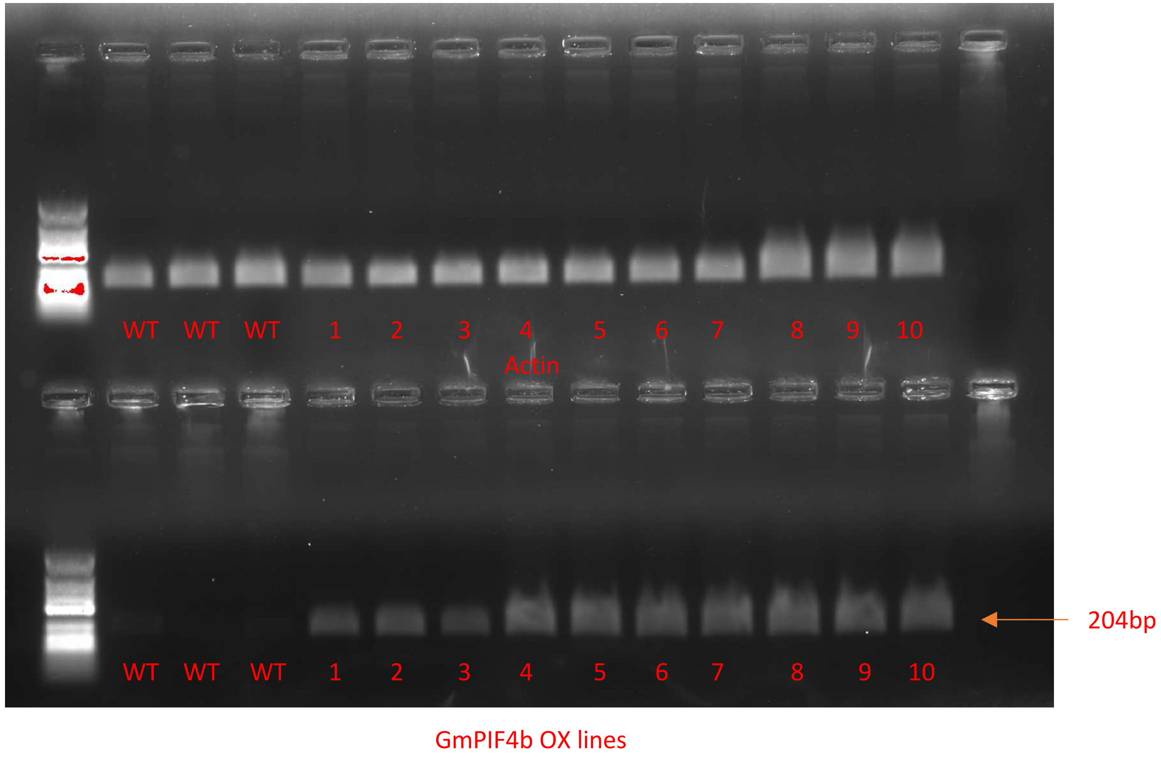

Supplementary Figure 5. Expression of Arabidopsis *actin* and *GmPIF4b* in over-expression lines *(35s::Gmpif4::polyA)*

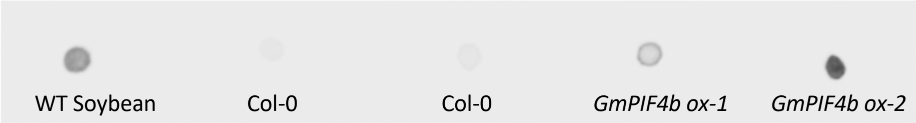

1/500 dilution of anti-GmPIF4b antibody

Supplementary Figure 6. Detection of GmPIF4 in transgenic plants using anti-GmPIF4b antibody in dot-blot assay- Total nuclear protein was extracted from soybean wild type plants, *Arabidopsis* wild type plants and two *GmPIF4b* over-expression lines of *Arabidopsis (GmPIF4b ox-1 and GmPIF4b ox-2)*. Anti–GmPIF4b antibody detected GmPIF4 in WT soybean and over expression *Arabidopsi*s lines but showed no reactivity with wild type *Arabidopsis* lines (i.e Col-0) .
