# Supplementary material for: Genomic and molecular analysis of conserved and unique features of soybean PIF4
Source: Sci Rep. 2018 Aug 22;8:12569. doi: 10.1038/s41598-018-30043-2 (PMC6105606; doi:10.1038/s41598-018-30043-2)
Supplement: Supplementary file 5 — Supplementary Dataset 4 [file 41598_2018_30043_MOESM5_ESM.docx]

| **Supplementary Table 4-A qRT-PCR primer information** | | |
| --- | --- | --- |
| Gene ID | Forward primer 5’-3’ | Reverse primer 5’-3’ |
| *Glyma.02G282100.1* | TGGTTCCCAAGCAGTGCAACAC | AAGGTGGAAGAACCCGCTTTGC |
| *Glyma.14G032200.1* | CTGTGGCAGCAGTCATATCC | TCTGATTTTCCTTTGTCACTCC |
| *Glyma.18G115700.1* | TATCCGCCGAGCCTGAAGCTAATG | AGCCACCGGATGATGAAGTTGTAG |
| *Glyma.13G130100.1* | AATGTCAGCAAGCCTAGTAGCAG | TGGCACTTTACCTGACTGATGC |
| *Glyma.02G160200.1* | AGATCTCATGCCGCAGAAGT | TGGTGGCATATACTGCTGGA |
| *Glyma.08G303900.1* | GGGCATCAAGCAATGGTGTTTGG | TCTCCTTAGCTTCTGGCTCGACTG |
| *Glyma.10G042800.1* | TGCTCCTCTGCTGCAATAGTAAGG | CTTCGTCGTCGTCGTCCATTTG |
| *GmFT2a* | GGATTGCCAGTTGCTGCTGT | GAGTGTGGGAGATTGCCAAT |
| *GmFT5a* | GCCTTACTCCAGCTTATACT | GGCATGCTCTAGCATTGCAA |
| *Arabidopsis actin (AT3G18780.2)* | TCTTCCGCTCTTTCTTTCCAAGC | ACCATTGTCACACACGATTGGTTG |
| *Glyma.08G146500.1 (Actin)* | ATCATGTTTGAGACCTTCAATGTG | CTCGAGTTCTTGCTCATAATCTAGG |
